# Anti-TMV Activity of Malformin A_1_, a Cyclic Penta-Peptide Produced by an Endophytic Fungus *Aspergillus tubingensis* FJBJ11

**DOI:** 10.3390/ijms16035750

**Published:** 2015-03-12

**Authors:** Qing-Wei Tan, Fang-Luan Gao, Fu-Rong Wang, Qi-Jian Chen

**Affiliations:** 1 Key Laboratory of Bio-pesticide and Chemistry-Biology, Ministry of Education, Fujian Agriculture and Forestry University, Fuzhou 350002, China; E-Mails: tanqingwei@fafu.edu.cn (Q.-W.T.); raindyok@126.com (F.G.); wfr1987@163.com (F.-R.W.); 2 Key Laboratory of Plant Virology of Fujian Province, Institute of Plant Virology, Fujian Agriculture and Forestry University, Fuzhou 350002, China

**Keywords:** malformin A_1_, cyclic peptide, *Tobacco mosaic virus* (TMV), *Aspergillus*, endophytic fungus, *Brucea javanica*

## Abstract

Plant-associated microorganisms are known to produce a variety of metabolites with novel structures and interesting biological activities. An endophytic fungus FJBJ11, isolated from the plant tissue of *Brucea javanica* (L.) Merr. (Simaroubaceae), was proven to be significantly effective in producing metabolites with anti-*Tobacco mosaic virus* (TMV) activities. The isolate was identified as *Aspergillus tubingensis* FJBJ11 based on morphological characteristics and ITS sequence. Bioassay-guided isolation led to the identification of a cycli penta-peptide, malformin A_1_, along with two cyclic dipeptides, cyclo (Gly-l-Pro) and cyclo (Ala-Leu). Malformin A_1_ showed potent inhibitory effect against the infection and replication of TMV with IC_50_ values of 19.7 and 45.4 μg·mL^−1^, as tested using local lesion assay and leaf-disc method, respectively. The results indicated the potential use of malformin A_1_ as a leading compound or a promising candidate of new viricide.

## 1. Introduction

*Tobacco mosaic viru*s (TMV) is one of the most well-studied plant viruses, which could infect more than 400 plant species belonging to 36 families, but until now no effective chemical treatments appeared capable of inhibiting virus replication and multiplication once it does infect host plants [1,2,3]. Plant-derived natural products represent a rich resource of novel structural and biological active leading compounds. Recent research revealed a series of natural products from plant, such as steroids, quassinoids, alkaloids, *etc.*, exhibited potent anti-TMV activity [3,4,5,6]. Plant-associated microorganisms are known to produce a variety of metabolites with novel structures and interesting biological activities, and the claimed medicinal properties and biological activities of some plant species have been attributed to the microorganisms living in association with these plants [7,8,9]. Therefore, it is a reasonable and promising approach to searching for potential biological active endophytic fungi from plants, which have been proved to be sources of antiviral metabolites.

With this in mind, we recently examined the anti-TMV activities of endophytic fungi isolated from the plant tissue of *Brucea javanica* (L.) Merr. (Simaroubaceae). *B. javanica* is a shrub widely distributed from Southeast Asia to Northern Australia. The seeds are used in Chinese folk medicine and contain predominant quassinoids, which have been proved to be the active anti-TMV components from the secondary metabolites of *B. javanica*. In the present work, one cyclic penta-peptide, along with two cyclic bipeptides, were isolated from the fermentation products of a fungal isolate under bio-assay guided isolation. We describe in this paper the identification of the active isolate, bio-assay guided isolation and structure elucidation of the active components.

## 2. Results

### 2.1. Identification of the Endophytic Fungus

**Figure 1:**
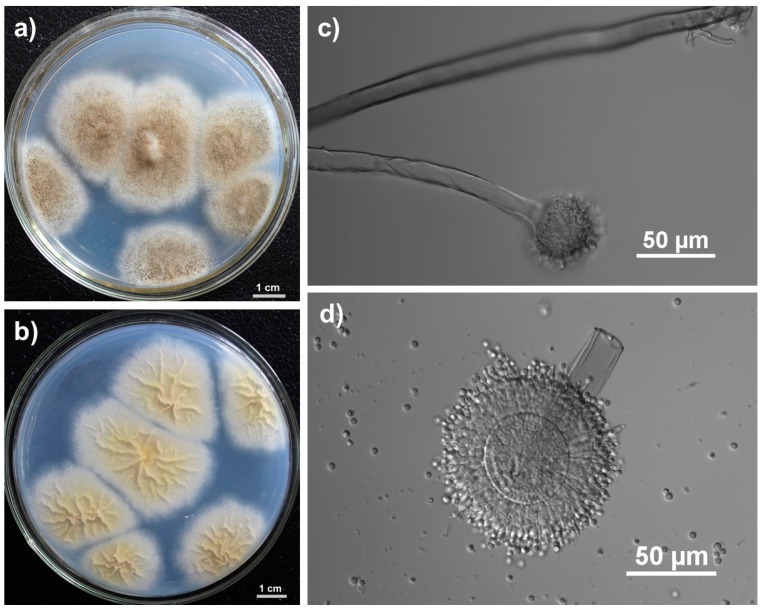
Morphological characteristics of isolate FJBJ11. (**a**) Colonies growing in Czapek’s agar for 7 days; (**b**) The yellowish colonies observed from the reverse side of the Czapek’s agar; (**c**) Sporophore and spherical sporangium; (**d**) Conidia and sporangium with bilayer structure.

Radial villus colonies began to form with white mycelia at the edge after growing on Czapek’s agar at 28 °C for three days. The colony became pitchy at the head of mycelium and yellowish in the reverse side after seven days. The hyphae were septate, mostly branched, 6–8 µm. Spherical sporangium was generated at the top of sporophore, and the typical bilayer structure was observed (Figure 1).

**Figure 2:**
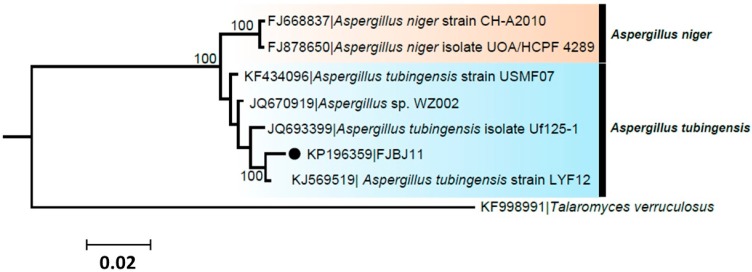
Phylogenetic relationship of isolate FJBJ11 (accession No. KP196359) to representative sequences of known *A. tubingensis* and *A. niger*. Reference sequences were retrieved from GenBank and a *T. verruculosus* strain (accession No. KF998991) was served as an outgroup. The numbers on the branches are posterior probability (only shown >50) supporting the branch pattern. The isolate FJBJ11 from current study is marked with black dot. The distance unit is substitutions/site.

Seven sequences of the genus *Aspergillus* downloaded from GenBank were divided into two groups as inferred from Bayesian phylogenetic inference. The isolate FJBJ11 (accession No. KP196359) was positioned in a group of *A. tubingensis* with high posterior probability support, while two strains of *A. niger* were clustered in another group (Figure 2). The isolate FJBJ11 shares the same common ancestor with *A. tubingensis* strain LYF12, which indicating it is a strain of *A. tubingensis*.

### 2.2. Fermentation, Bio-Assay Guided Isolation, and Antiviral Activity of the Active Component

The endophytic fungus strain, *Aspergillus tubingensis* FJBJ11, was fermented in liquid Sabouraud medium. Macroporous absorption resin was employed to obtain a crude extract, and then diluted in MeOH to afford a MeOH soluble fraction, which was proved to be the active fraction. The active fraction showed potent inhibition against the infection and replication of TMV at a test concentration of 1 mg·mL^−1^, with inhibitory rates of 97.2% and 88.6% as determined by using local lesion assay and leaf-disc method, respectively.

**Figure 3:**
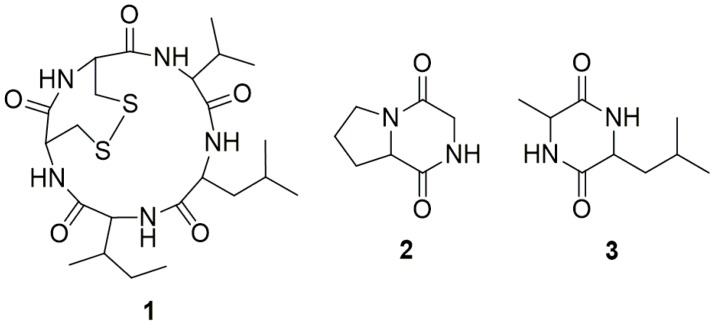
Chemical structures of the cyclic peptides **1**–**3**.

**Figure 4:**
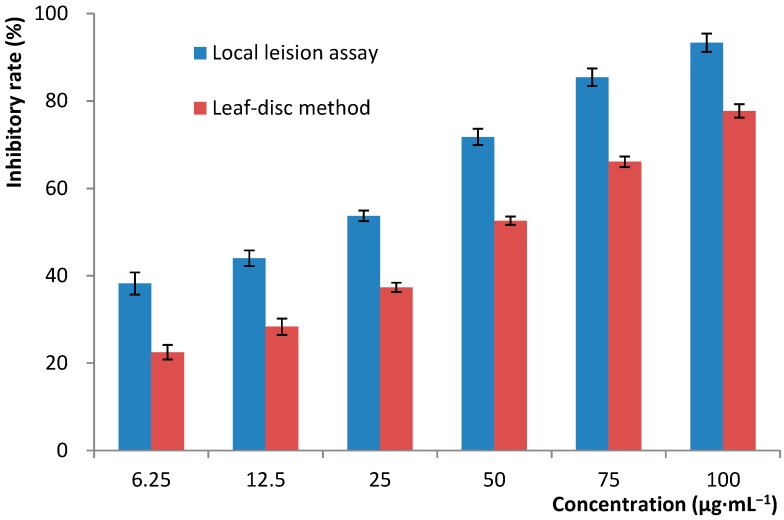
Dose-dependent inhibition of malformin A_1_ against the replication of TMV as tested by using local lesion assay and leaf-disc method. Error bar: ±SD.

Glucan gel column chromatography was then used as the sole method to separate and purify the active constituents. The active MeOH soluble fraction was first subjected to column chromatography with Sephadex LH-20 eluted with 80% MeOH to give six fractions, Fr.1–Fr.6, among which only Fr.2 showed potent anti-TMV effect with an inhibitory rates of 98.7% (at a concentration of 500 μg·mL^−1^) as tested through leaf-disc method. Other fractions were non-active with inhibitory rates no more than 15% under the same conditions. Fr.2 was then purified by Sephadex LH-20 column chromatography eluted with a mixture of CHCl_3_ and MeOH (*v*/*v*, 1:1) to give three cyclic peptides, malformin A_1_ (**1**), cyclo (Gly-l-Pro) (**2**), and cyclo (Ala-Leu) (**3**) (Figure 3). Compounds **2** and **3** were proved to be non-active (inhibitory rates < 15% at a concentration of 200 μg·mL^−1^). Malformin A_1_ (**1**) was finally determined to be the main and sole active anti-TMV constituent produced by the endophytic fungus strain, *A. tubingensis* FJBJ11. The IC_50_ values were calculated to be 19.7 and 45.4 μg·mL^−1^ according to the data obtained through local lesion assay and leaf-disc method (Figure 4), respectively. The positive control, ningnamycin, possessed IC_50_ values of 68.7 and 185.2 μg·mL^−1^ as tested under the same condition.

### 2.3. Structure Determination

The structure of the active component, malformin A_1_ (**1**), was elucidated based on the analysis of the 1D and 2D NMR data, as well as by comparing the NMR data with those in the existing literature. The NMR spectra of **1** (Table 1) showed five amide proton signals (δ_H_ 7.11 (d, *J* = 11.0 Hz), 7.38 (d, *J* = 9.2 Hz), 7.94 (d, *J* = 8.5 Hz), 8.59 (d, *J* = 6.6 Hz), 8.84 (d, *J* = 3.7 Hz)) and five carbonyls (δ_C_ 169.8, 170.6, 172.8, 173.0, 174.0), which indicated that **1** might belong to the peptide class of compounds. The amino acid residues and the connectivity were deduced from the analysis of 2D NMR data (HSQC, ^1^H–^1^H COSY, and HMBC). The NMR data were consistent with those in the literature [10,11]. Thus, compound **1** was identified as malformin A_1_ (cyclo-Leu^1^-Ile^2^-Cys^3^-Cys^4^-Val^5^). The structures of other two known compounds, cyclo (Gly-l-Pro) (**2**), cyclo (Ala-Leu) (**3**), were elucidated based on their physicochemical properties and spectral data, as well as by comparison of the NMR data with those in the existed literature.

**Table 1:** NMR data of malformin A_1_ (**1**).

Amino Acid	Position	δ_H_ (mult., *J* in Hz)	δ_C_ (mult.)
Leu^1^	C=O	–	172.8 (s)
	NH	7.38 (d, 9.2)	–
	α	4.47 (dt, 9.2, 2.8)	50.4 (d)
	β	1.37 (m)	40.9 (d)
	γ	1.56 (m)	24.5 (d)
	δ	0.89 (d, 6.6)	22.7 (q)
		0.85 (d, 6.6)	21.8 (q)
Ile^2^	C=O	–	173.0 (s)
	NH	8.59 (d, 6.6)	–
	α	3.87 (dd, 10.2, 6.6)	58.0 (d)
	β	1.69 (m)	34.0 (d)
	γ	1.50 (m); 1.13 (m)	24.8 (t)
		0.77 (d, 6.9)	14.9 (q)
	δ	0.81 (t, 10.9)	10.0 (q)
Cys^3^	C=O	–	174.0 (s)
	NH	8.84 (d, 3.7)	–
	α	3.98 (dd, 6.3, 3.3)	52.4 (d)
	β	3.51 (dd, 11.9, 3.2); 3.14 (m)	46.2 (d)
Cys^4^	C=O	–	169.8 (s)
	NH	7.11 (d, 11.0)	–
	α	4.71 (dt, 11.0, 4.4)	52.9 (d)
	β	3.24 (m); 3.19 (m)	45.2 (t)
Val^5^	C=O	–	170.6 (s)
	NH	7.94 (d, 8.5)	–
	α	3.92 (m)	58.8 (d)
	β	2.05 (m)	26.9 (d)
	γ	0.82 (d, 6.8)	19.7 (q)
		0.82 (d, 6.8)	18.7 (q)

## 3. Discussion

Malformin A_1_ (**1**), produced from *Aspergillus niger*, is a cyclic pentapeptide with a disulfide bond formed from two cysteine thiols. Malformin A_1_ has been reported to possess a variety of biological activities including plant growth stimulation [12], phytochrome-mediated response modulation of *Phaseolus vulgaris* [13], antibacterial activity [14,15], prevention of interleukin-1 (IL-1) induced endothelial changes by inhibition of protein synthesis [16], as well as enhanced fibrinolytic activity [17]. Moreover, malformin A_1_ was recently found to be strongly cytotoxic against the human cancer cell lines NCI-H460 (non-small cell lung carcinoma), MIA Pa Ca-2 (pancreatic cancer), MCF-7 (breast cancer), and SF-268 (CNS cancer; glioma) with slight selectivity towards the pancreatic cancer cell line (MIA Pa Ca-2) compared with the normal human primary fibroblast cells WI-38 [18]. Another research indicated that malformin A_1_ exhibit quite significant cytotoxic activities against the human A2780, H1688, K562, M231, PC3 cell lines [19].

Bio-assay guided isolation indicated that malfomin A_1_ was the main and sole active component produced by *A. tubingensis* FJBJ11 in our current study. Malfomin A_1_ could be obtained efficiently by fermentation, and a simple separation and purification procedure has been promoted in current study. Malformin A_1_ possesses a potent inhibitory effect against the infection and replication of *Tobacco mosaic virus* (TMV) as determined through local lesion assay and leaf-disk method. The results suggest a potential utilization of malformin A_1_ as natural viricide, or otherwise as leading compound for the development of novel antiviral agents. The interaction between tobacco and TMV was a traditional, frequently-used and effective system for selection of novel antiviral candidates. The scientific value of such research could be amplified if much more target phytopathogenic viruses, even animal or human viruses, were considered. The various and significant biological activities of malfomin A_1_ are drawing more and more scientific interest, while the current results indicate that much more attention is worthy of being put on its antiviral property.

The malformin complex, which comprises a small family of cyclic penta-peptides, was first presented as a plant growth regulator that evoked the malformation of stems and petioles. Malformin is closely related to ethylene production, and has been suggested that the organ-specific growth disturbances may be mediated, in part, by malformin-modulated ethylene production. Research indicated that malformin A_1_ has dose-dependent effects on auxin/indole acetic acid (IAA)-induced ethylene production in mung bean. Malformin A_1_ can modulate ethylene production through diverse paths and its effect depends on the concentration of the treatment administered [20].

The role of ethylene in the hormonal regulation of plant development has been well established, and it has become clear that ethylene plays an important role in mediating different types of induced resistance. Induction of plant resistance, either achieved by chemicals (systemic acquired resistance, SAR), or by rhizobacteria (induced systemic resistance, ISR) is a possible and/or complementary alternative to manage virus infections in crops. Both ISR and SAR are a condition of alerted defense that provides long-lasting, broad-spectrum resistance, which is effective against different pathogens, including viruses. SAR mechanisms operating against viruses are diverse, depending on the pathosystem, and may inhibit virus replication as well as cell-to-cell and long-distance movement. Studies have indicated an important role of ethylene in establishing SAR in tobacco against TMV [21,22]. Based on such facts, the antiviral properties of malformin A_1_ could possibly be due to, at least in part, its abilities of modulating the ethylene production, which in turn elicited defense mechanisms of host plant.

In recent years, ethylene have been shown to act synergistically with auxin, another well studied hormone, to control specific growth and developmental processes, such as root elongation and root hair formation, as well as antagonistically in other processes, such as lateral root formation and hypocotyl elongation. An emerging trend is that ethylene modulates auxin synthesis, transport, and signaling with unique targets and responses in a range of tissues to fine-tune seedling growth and development. Moreover, recent studies suggested that TMV replicase-Aux/IAA protein interactions selectively enhance virus pathogenicity in tissues where Aux/IAA proteins accumulate, *viz*., the TMV replicase protein interferes with the plant’s auxin response system to induce specific disease symptoms [23,24,25]. Thus, the potential effect of malfomin on auxin biosynthesis should also be considered in future attempt to unravel their physiological effects on plant growth, as well as to elucidate the underlying mechanisms of the antiviral properties of malformin A_1_.

## 4. Experimental Section

### 4.1. General

The NMR spectra were obtained by using a Bruker AV-400 or a DRX-500 spectrometer. Mass spectra were recorded on a Jeol JMS-HX 110 instrument. Sephadex LH-20 (25–100 μm, Pharmacia Fine Chemical Co., Ltd., Uppsala, Sweden), Silica gel (200–300 mesh), and Silica gel H (Qingdao Oceanic Chemical Co., Qingdao, China) were used for column chromatography. Thin-layer chromatography was performed on TLC plates (Qingdao Oceanic Chemical Co., Qingdao, China), with compounds visualized with iodine vapor. Isolate cultured on Czapek’s agar at 28 °C for 7 days was used for microscopic observation, with images acquired using a Nikon TiE System (Nikon, Tokyo, Japan).

### 4.2. Bioassay of TMV-Inhibitory Activity

Local lesion assay and leaf-disc method were employed to determine the TMV-inhibitory activities as previously described [26,27]. *Nicotiana tabacum* cv. K326 and *Nicotiana glutinosa* L., used as systematic and local lesion hosts of *Tobacco mosaic virus* (TMV), respectively, were cultivated in an insect-free glasshouse at 25 °C. Plants of 5–6-leaf stage were used for experiments. TMV (U1 strain) was propagated and maintained in the systematic host, *N. tabacum* cv. K326. Each sample was dissolved in DMSO and diluted with water to provide test solutions, with a final concentration of DMSO less than 0.5%. Ningnanmycin, a commercial antiviral agent, was used as positive control. A solution of DMSO with equal concentration was used as negative control.

#### 4.2.1. Local-Lesion Assay

A mixture of test solution with TMV (final concentration of 10 μg·mL^−1^) was made 30 min before inoculation. The mixture was inoculated on the left side of the leaves of *N. glutinosa*, while the right side of the same leaves were inoculated with solution of negative control mixed with equal concentration of TMV. All assays were conducted in triplicate. The numbers of local lesion were recorded 3–4 days after inoculation, and inhibition rates were calculated according to the following formula:

Inhibition rate (%) = [(*C* − *T*)/*C*] × 100% (1)

where *C* is the average local lesions number of the control, while *T* is the average local lesions number of the treatment.

#### 4.2.2. Leaf-Disc Method

Leaves of *N. tabacum* cv. K326 were mechanically inoculated with TMV solution (10 μg·mL^−1^). Leaf discs of 1 cm in diameter were punched 8 h after the inoculation, and floated on the solution of each sample and the negative control at 25 °C for 48 h. Leaf discs were then ground in coating buffer (sodium carbonate buffer, pH 9.6), followed by enzyme-linked immunosorbent assay (ELISA). Six repetitions of each treatment were conducted. The virus concentrations were calculated, according to the OD_405_ values obtained, by standard curve, which was established with a series of purified virus solutions of different concentrations. The inhibition rates of virus replication were calculated according to the following formula:

Inhibition rate (%) = (1 − *T*_0_/*C*_0_) × 100% (2)

where *T*_0_ is the virus concentration in the treated leaf discs, while *C*_0_ is the virus concentration in the leaf discs of the control.

#### 4.2.3. Indirect ELISA Procedure

Each well of a micro-ELISA plate was filled with 100 μL of diluted antigen and incubated at 4 °C for 24 h. The antigen solution was then discarded and each well was washed three times by using phosphate-buffered saline (PBS, pH 7.2), containing 0.001% Tween 20 (PBS-T); Secondly, rabbit anti-TMV serum (1:2000) was added to the antigen-coated wells and incubated at 37 °C for 1 h, and then washed three times using PBS-T; Thirdly, goat anti-rabbit alkaline phosphatase conjugate (1:30,000) in PBS-T was added, and incubated at 37 °C for 1 h, followed by washing three times with PBS-T; Finally, 100 μL of *p*-nitrophenyl phosphate (PNP) substrate (1 mg·mL^−1^) was added per well, and, 10 min later, the reaction was stopped by adding 100 μL of 3 M sodium hydroxide. The intensity of color development was determined by measuring absorbance with a micro-ELISA reader equipped with a 405 nm filter.

#### 4.2.4. Statistical Analysis

All data were stored and analyzed with the SPSS 19.0 software program (IBM, New York, NY, USA).

### 4.3. Identification of Fungi

Filamentous fungi were identified to genus and species level, respectively, by examining the culture morphology and by microscopy, as well as with molecular methods. DNA was extracted from mycelia with the Plant Genomic DNA Purification Kit (Tiangen Biotech Co., Ltd., Beijing, China). PCR amplification was conducted with the forward primer ITS1 (5'-TCCGTAGGTGAACCTGCGG-3') and reverse primer ITS4 (5'-TCCTCCGCTTATTGATATGC-3') for the ITS region as described by White *et al.* [28]. Sequencing was performed by Life Technologies (Shanghai, China).

Fungal identification was performed in GenBank by using the BLAST program and the maximum identity as the first identification criteria. Multiple sequence alignments were performed with MUSCLE implemented in MEGA5 [29]. Phylogeny inference was reconstructed with the Bayesian inference (BI) performed in MrBayes version 3.22, which uses Markov chain Monte Carlo (MCMC) simulation to estimate posterior distributions [30]. The Hasegawa–Kishino–Yano (HKY) model determined by MrModelTest was used to reconstruct phylogenetic tree as the model fits best to the data [31]. Bayesian analyses achieved stationary in 2 × 10^6^ generations, which was assessed by the average standard deviation of split frequencies. The recommended average standard deviation on split frequency value should be below 0.01.

### 4.4. Endophytic Fungus and Fermentation

The endophytic fungal isolate FJBJ11 was isolated from the healthy stems of *Brucea javanica* (L.) Merr. (Simaroubaceae) collected in Xiamen Overseas Chinese Subtropical Plant Introduction Garden (Xiamen, China). The strain is deposited in the Key Laboratory of Bio-pesticide and Chemistry-Biology, Ministry of Education, Fujian Agriculture and Forestry University, Fuzhou, China. The isolate was cultured in 500 mL Erlenmeyer flasks each containing 200 mL of potato dextrose broth (PDB) medium at 25 °C on a rotary shaker at 120 rpm for 8 days to obtain the seed culture. Cultivation was carried out in a 50 L fermentor containing 30 L of liquid Sabouraud medium at 25 °C and 120 rpm for 15 days.

### 4.5. Extraction and Isolation

The fermentation broth (30 L) was harvested and filtrated. The supernatant was desalted by Diaion HP20 column eluted with H_2_O, and then eluted with MeOH to give 80 g of crude extract, which was then diluted in 1 L of MeOH and filtered. The MeOH soluble fraction (37 g) was subjected to column chromatography with Sephadex LH-20 eluted with 80% MeOH to give six fractions, Fr.1–Fr.6. The only active fraction, Fr.2 (500 mg), was subsequently subjected to Sephadex LH-20 eluted with a mixture of CHCl_3_ and MeOH (*v*/*v*, 1:1) to give compounds **1** (260 mg), **2** (73 mg), and **3** (159 mg).

#### 4.5.1. Malformin A_1_ (**1**)

Amorphous white powder. ^13^C NMR (101 MHz, DMSO) δ 174.0, 173.0, 172.8, 170.6, 169.7, 58.8, 58.0, 52.9, 52.4, 50.3, 46.2, 45.2, 40.9, 34.0, 26.8, 24.7, 24.5, 22.7, 21.8, 19.7, 18.7, 14.9, 10.0. ^1^H NMR (400 MHz, DMSO) δ 8.84 (d, *J* = 3.7 Hz), 8.59 (d, *J* = 6.8 Hz), 7.94 (d, *J* = 8.6 Hz), 7.38 (d, *J* = 9.2 Hz), 7.11 (d, *J* = 10.8 Hz), 4.71 (dt, *J* = 10.8, 4.3 Hz), 4.46 (dt, *J* = 9.2, 6.2 Hz), 3.98 (dd, *J* = 6.5, 3.7 Hz), 3.92 (t-like, *J* = 9.6 Hz), 3.86 (dd, *J* = 6.8, 3.6 Hz), 3.51 (dd, *J* = 11.8, 3.2 Hz), 3.24 (m), 3.21 (m), 3.14 (m), 2.03 (m), 1.68 (m), 1.56 (m), 1.50 (m), 1.37 (m), 1.14 (m), 0.89 (d, *J* = 6.6 Hz), 0.85 (d, *J* = 6.6 Hz), 0.83 (d, *J* = 6.8 Hz), 0.81 (t, *J* = 10.9 Hz), 0.81 (d, *J* = 6.8 Hz), 0.77 (d, *J* = 6.8 Hz). ESIMS *m*/*z* 530.2 [M + H]^+^.

#### 4.5.2. Cyclo (Gly-l-Pro) (**2**)

Amorphous white powder. ^13^C NMR (101 MHz, DMSO) δ 169.4, 164.0, 58.1, 45.9, 44.7, 27.9, 22.1. ^1^H NMR (400 MHz, DMSO) δ = 8.07 (s), 4.12 (m), 4.01 (br.s), 3.97 (br.s), 3.53 (d, *J* = 4.6 Hz), 3.49 (d, *J* = 4.6 Hz), 3.33 (m), 2.12 (m), 1.80 (m). ESIMS *m*/*z* 155.1 [M + H]^+^.

#### 4.5.3. Cyclo (Ala-Leu) (**3**)

Amorphous white powder. ^13^C NMR (101 MHz, DMSO) δ = 169.0, 168.8, 52.6, 50.0, 42.7, 23.7, 23.0, 21.9, 19.7. ^1^H NMR (400 MHz, DMSO) δ 8.13 (s), 8.10 (s), 3.86 (m), 3.76 (m), 1.81 (m), 1.60 (m), 1.46 (m), 1.26 (d, *J* = 7.0 Hz), 1.05 (m), 0.87 (d, *J* = 6.6 Hz), 0.85 (d, *J* = 6.6 Hz). ESIMS *m*/*z* 185.1 [M + H]^+^.

## 5. Conclusions

Malformin A_1_, a cyclic penta-peptide produced from *Aspergillus* fungus, is proven to be a highly efficient inhibitor of *Tobacco mosaic virus* (TMV) infection and replication, which indicated the potential use of malformin A_1_ as a leading compound or a promising candidate of a new viricide.
